# Occurrence of Glyphosate and Other Polar Pesticides in Honey from Lombardy and Emilia-Romagna Regions in Italy: Three-Year Monitoring Results

**DOI:** 10.3390/foods12244448

**Published:** 2023-12-12

**Authors:** Elena Butovskaya, Mara Gasparini, Barbara Angelone, Gabriella Cancemi, Vito Tranquillo, Giovanni Prestini, Filippo Bosi, Simonetta Menotta

**Affiliations:** 1 Food and Feed Chemistry Department, Istituto Zooprofilattico Sperimentale della Lombardia e dell’Emilia Romagna “Bruno Ubertini” (IZSLER), via A. Bianchi 9, 25124 Brescia, Italy; mara.gasparini@izsler.it (M.G.); barbara.angelone@izsler.it (B.A.); gabriella.cancemi@izsler.it (G.C.); simonetta.menotta@izsler.it (S.M.); 2 Programmazione dei Servizi e Controllo di Gestione, Istituto Zooprofilattico Sperimentale della Lombardia e dell’Emilia Romagna (IZSLER), via A. Bianchi 9, 25124 Brescia, Italy; vito.tranquillo@izsler.it; 3 Dipartimento Veterinario e Sicurezza degli Alimenti di Origine Animale, ATS della BRIANZA, Viale Elvezia 2, 20900 Monza, Italy; giovanni.prestini@ats-brianza.it; 4 Dipartimento di Sanità Pubblica, Azienda Unità Sanitaria Locale della Romagna–Ravenna, via Fiume Montone Abbandonato 134, 48100 Ravenna, Italy; filobosivet@gmail.com

**Keywords:** polar pesticides, glyphosate, pesticide residues, IC-HRMS, honey

## Abstract

Intensive agricultural practices, such as pesticides use, may negatively affect bee health and hive products. Glyphosate is one of the most widely used polar pesticides applied in crops for weed control. In this study, honey samples, collected from beekeeping farms located in the Lombardy and Emilia-Romagna regions in Italy in the framework of regional monitoring plans activated from 2020 to 2022, were analyzed for the presence of residues of polar pesticides. The analytical method based on ion chromatography coupled to high-resolution mass spectrometry was applied to quantify glyphosate, glufosinate, ethephon, fosetyl aluminum, and their related metabolites. Residues of glyphosate were detected in around 28% of analyzed honey samples. Observations on the distribution of the honey-production-site locations suggest that honey samples originating from the provinces within the Lombardy region, where the agricultural sector is highly developed, were more affected by glyphosate contamination than the samples collected from the areas with low agricultural activity, where no glyphosate residues were detected over the three years of the monitoring program.

## 1. Introduction

Honey is a natural hive product consumed worldwide. The European Union is the second largest honey producer after China, with 220,000 tons yielded in 2021, and Italy is estimated to account for approximately 7% of European honey production annually [1]. In Northern Italy, the Lombardy and Emilia-Romagna regions share 11.5% and 10.6% of the total national apiaries, respectively [2]. Along with important production yields, the beekeeping sector is essential for agriculture, as it helps to preserve the bee population crucial for pollination of crops intended for food production, and generally ensures plant reproduction, diversity, and abundance [3]. On the other hand, while being very important in the agricultural sector, bees, and consequently the quantity and the quality of derived products, may be negatively affected by intensive agricultural practices, such as use of pesticides [4]. Bees may come into contact with the pesticides while foraging on directly treated crops or through contaminated environmental sources, such as water and soil [5]. Pesticides may accumulate in the hive, resulting in the presence of their residues in edible hive products (for instance, honey, pollen, and royal jelly) and posing a potential risk for consumers. Due to the constant connection to the environment surrounding the apiaries, bees and hive products are naturally exposed to the environmental contaminants and have been proposed as bioindicators of environmental pollution [6]. Their potential role in biomonitoring has been described in both industrialized and agricultural areas, where the environmental contamination with heavy metals and with pesticides has been investigated through the occurrence in hive products [7,8,9].

Glyphosate (GLY) is a systemic, non-selective herbicide intensively used in crop fields worldwide [10]. According to Benbrook et al., around 746,580 tons of GLY was applied globally in the agricultural sector in 2014 [11], while Antier et al. estimated that 49,427 tons of the herbicide was sold in Europe in 2017 [12]. Together with the increased trends in GLY consumption, concerns have been raised about its possible toxic effects on human health and environmental impact [13]. GLY was classified as “probably carcinogenic to humans” (Group 2A) by IARC (International Agency for Research on Cancer) in 2015 [14], while EFSA (European Food Safety Authority) concluded that “glyphosate is unlikely to pose a carcinogenic hazard to humans” [15]. The Committee for Risk Assessment of European Chemicals Agency (ECHA) classified glyphosate as causing serious eye damage and being toxic to aquatic life, while concluding again in 2022 that classifying glyphosate as a carcinogen is not justified [16]. GLY is currently approved in the European Union until 15 December 2033. It is applied in annual cropping systems to control weeds before and during cultivation, in perennial tree crops, and in grasslands for several purposes, such as terminating temporary grasslands or renewal of permanent ones [12]. Besides the predominant use in the agricultural sector, GLY is also employed to kill weeds in industrialized areas, alongside railways, roadsides, and home gardens. 

The Regulation (EC) 396/2005, amended by the Commission Regulation (EU) 293/2013, established maximum residue limits (MRLs) of pesticides for food commodities of plant and animal origin, setting the MRL at 0.05 mg kg^−1^ for GLY residues in honey [17,18]. Additionally, in Italy, the MRL of 0.01 mg kg^−1^ is set for the pesticides (GLY included) in foods from organic agriculture [19]. 

GLY belongs to the group of polar pesticides, which includes a variety of compounds, such as glufosinate, fosetyl aluminum, ethephon, and their related metabolites, characterized by similar physicochemical properties: small molecular size, high polarity, and high water solubility [20]. These properties make the analytical procedures for their detection at trace level in food matrices extremely challenging, and the common multiresidue methods used for pesticide determination are not suitable due to extraction and purification issues [21]. Generally, chromatographic separation, followed by tandem-mass-spectrometry (MS/MS) detection, is applied for polar-pesticide group analysis or single-residue determination [22,23,24]. Recently, alternative methodologies based on ion chromatography coupled to high-resolution mass spectrometry (IC-HRMS) have been developed to overcome chromatographic challenges and combine the determination of multiple highly-polar-pesticide residues, including GLY, in a single analysis [25,26].

After newly developed methodologies were made available, in the Lombardy and Emilia-Romagna regions in Italy, the monitoring of honey for the presence of polar pesticides, including GLY, was activated in the framework of official regional monitoring programs starting from 2020. In the context of food-safety purposes, the monitoring program also aimed to give insights about the environmental pollution of local territories with pesticides. Therefore, in this study, honey samples from two Italian regions were analyzed in the framework of regional monitoring programs from 2020 to 2022. Furthermore, a focus was made on the GLY residues in honey samples, and monitoring results were related to the agricultural-activity extension and the types of crops harvested in the different provinces of two regions in order to evaluate a possible correlation between honey contamination with the herbicide and the agricultural activities developed in the considered provinces.

## 2. Materials and Methods

### 2.1. Sample Collection

A total of 221 samples were collected from honey production sites located in the Lombardy and Emilia-Romagna regions in the framework of official regional monitoring plans implemented during the years 2020–2022. According to the Commission Directive 2002/63/EC, establishing community methods of sampling for the official control of pesticide residues in and on products of plant and animal origin [27], at least 100 g of honey were collected directly from the hive or from the honey extraction laboratories. Sampling was performed from May to October of each year. Precisely, during 2020, 2021, and 2022, in the Lombardy region, 11, 13, and 45 honey samples were collected directly from hives and 38, 42, and 6 from extraction laboratories, respectively. In Emilia-Romagna, the total number of samples per year was 22, with 5, 1, and 4 samples collected from hives during 2020, 2021, and 2022, respectively. All samples were stored at room temperature prior to analysis. 

### 2.2. Sample Preparation and IC-HRMS Analysis

Honey samples were subjected to IC-HRMS analysis for the quantification of 4 polar-pesticide residues and related metabolites (glyphosate, N-acetyl glyphosate, aminomethylphosphonic acid (AMPA), N-acetyl AMPA, glufosinate, 3-methylphosphonicpropionic acid (MPPA), N-acetyl glufosinate (NAG), ethephon, ethephon hydroxy, fosetyl aluminum, and phosphonic acid) by the analytical method validated according to the SANTE/11312/2021 criteria [26], previously published by [24]. Quantification limits (LOQs) were as follows: 0.01 mg kg^−1^ for glyphosate, N-acetyl glyphosate, N-acetyl AMPA, glufosinate, MPPA, NAG, and ethephon hydroxy; 0.05 mg kg^−1^ for ethephon and AMPA; and 0.1 mg kg ^−1^ for fosetyl aluminum and phosphonic acid. Briefly, 2.5 g of homogenized honey sample was weighed and extracted with 10 mL of water acidified with 1% formic acid and 10 mL of water/acetonitrile solution (9:1 *v*/*v*). After the extraction step, 2.5 mL of each sample was filtered on HLB Prime Cartridge with 335 mg of sorbent content (Waters, Milford, MA, USA). After sample dilution, 50 µL of purified extracts was injected into the IC-HRMS system. Blank and spiked samples (LOQ concentration for each analyte) were processed in the same conditions for each analytical batch. Labelled internal standards were added to each sample to trace the extraction efficiency and matrix effect. Instrumental analysis was performed with the Dionex IC 5000+ (Thermo Fischer Scientific, Waltham, MA, USA) ion chromatograph coupled with the Q-Exactive Focus mass spectrometer (Thermo Fischer Scientific, Waltham, MA, USA), following the chromatographic and spectrometric conditions previously described by [26]. Analytes were quantified against their isotopically labelled analogues, calculating the mean response factor obtained from the injection of six standard solution concentrations ranging from 0.2 times LOQ to 5 times LOQ. If the concentration of the analytes was close to the MRL, the honey sample was reprocessed in duplicate; the final result was expressed as the mean of the obtained values. The uncertainty calculation of 50% was applied for the compliancy evaluation [28].

### 2.3. Evaluation of Agricultural Activity and Use of Herbicides in Lombardy and Emilia-Romagna Regions

The data on the herbicide use and areas dedicated to the cultivations were obtained from datasets available on the ISTAT website [29]. The average use of herbicides per province was calculated as a ratio between the kilograms of herbicides (active ingredients) distributed in each province annually (average from 2020 and 2021) and the hectares within the province dedicated to the cultivations (all types of crops reported in the dataset for years 2020 and 2021). For annual crop classification, different types of cereals and leguminous plants were included, while for perennial crops, arboreal cultivations were considered. Permanent and temporary grasslands were considered in the pastures-and-meadows category.

### 2.4. Statistical Analysis

The correlations between the percentage of cultivated lands within the considered territories and the contamination rates with GLY residues were estimate with Spearman’s non-parametric, rank-based correlation coefficient Rho with 95% Confidence Intervals computed by boostrapping using the spearman.ci function of the RVAideMemoire package [30] in the R language [31]. To visualize the relationship between two variables in a dataset and to identify any trends or patterns we use scatterplot with add regression line with standard error bands using the ggplot function of the ggplo2 R package [32].

## 3. Results

### 3.1. Residues Detected in Honey Samples

The following pesticides were detected during the analysis of honey samples: fosetyl aluminum, phosphonic acid (metabolite of fosetyl aluminum), and GLY. Residues of phosphonic acid were detected in 15 samples prevalently in the Emilia-Romagna region (13 samples), and in the majority of cases (11 samples), the presence of phosphonic acid was associated with the presence of GLY residues, while only in one case it was associated with its parent fosetyl aluminum and GLY residues. GLY was the most frequently detected analyte in honey samples. Therefore, the focus of the following results is on the glyphosate residues.

Residues of GLY were detected in 62 of 221 honey samples (28%). In the Lombardy region, the contamination rate remained stable over the years (23.5%, 23.6%, and 24.0%), while in the Emilia-Romagna region, 12 of 22 (54.4%) analyzed samples resulted in detectable GLY residues in 2020, decreasing to 6 and 7 samples with detectable GLY residues (the total number of analyzed samples remained constant) during the two following years, respectively (Table 1). 

In the Lombardy region, the Mantua and Cremona provinces had the highest percentage of detected GLY residues over the years, with 53.8% (n = 13) and 55.5% (n = 9) positivity rate, respectively, while in the Sondrio, Como, and Varese provinces, no residues were detected during the three-year monitoring (Table 1). In the Monza and Brianza province, only two samples, where no GLY residues were detected, were analyzed during the monitoring program, and the province was not taken into consideration for further analysis.

In the Ferrara, Ravenna, Modena, and Piacenza provinces within the Emilia-Romagna region, the positivity rates reached 72.7% (n = 11), 75% (n = 4), 80% (n = 5), and 83.3% (n = 6), respectively. In Forlì-Cesena, Parma, and Rimini, no GLY residues were detected (Table 1).

During the monitoring years, GLY residues were detected twice at concentrations exceeding the established MRL of 0.05 mg kg^−1^. Precisely, 0.31 mg kg^−1^ and 0.25 mg kg^−1^ levels, around five-fold MRL, were detected in Emilia-Romagna (Ferrara province) and Lombardy (Cremona province), respectively. Six samples from the Lombardy region and two samples from Emilia-Romagna displayed GLY concentrations close to the MRL but were considered compliant due to the application of the uncertainty calculation (Table 1). Bergamo and Lodi registered mean concentrations close to the MRL value, 0.041 mg kg^−1^ (n = 4) and 0.044 mg kg^−1^ (n = 2), respectively. In Cremona, the mean concentration arrived at 0.078 mg kg^−1^ (n = 5), above the MRL concentration (due to the contribution of the non-compliant sample). In Emilia-Romagna, the Ravenna province reached a 0.04 mg kg^−1^ (n = 3) mean concentration value, while Ferrara stayed above the 0.05 mg kg^−1^ level with a mean concentration of 0.058 mg kg^−1^ (n = 8) due to the contribution of the non-compliant sample. In three years, 24 collected samples came from production sites with organic management systems, and GLY residues were detected in 5 of them. Four samples came from Lombardy and one from Emilia-Romagna (only four samples from organic apiculture were collected from this region over the three years). The concentration of detected GLY residues ranged from 0.01 mg kg^−1^ to 0.032 mg kg^−1^. According to the Regulation (EU) 2018/848 on organic production, apiaries shall be kept at sufficient distance from sources that may lead to the contamination of apiculture products and within a radius of 3 km from the apiary site; nectar and pollen sources shall consist essentially of organically produced crops or spontaneous vegetation or crops treated with low-environmental-impact methods [33].

Our results suggest that honey from apiaries managed with organic production systems is also subject to contamination with the herbicide GLY. Chiesa et al. reported low levels of contamination with the organochlorine and organophosphate pesticides in organic honeys from different Italian regions where citrus and apple orchards are substantial parts of developed agricultural systems [34], while no traces of GLY were detected in organic honeys from the Apulia region [35]. However, further investigations including higher numbers of organic samples are needed to draw consistent conclusions. 

### 3.2. Average Use of Herbicides in Lombardy and Emilia-Romagna Regions

As described above, monitoring results displayed the nonhomogeneous pattern of detectable GLY residues within the regions, suggesting that data may be affected by the amounts of GLY used in agricultural practice. Unfortunately, the specific data on the GLY use on the regional level were not available. However, the category “herbicides”, where GLY can be identified as a substantial part (GLY accounted for 52% of the herbicides sold in Italy in 2017, according to Antier et al. [12]), was considered. Therefore, the average use of herbicides per province was calculated as the rate of the herbicides’ amount, defined as kilograms of active ingredients, and hectares dedicated to the agricultural activity (kg a.i./ha). In the Lombardy region, the estimated average use of herbicides among the provinces resulted in nonuniformity (Table 2). Sondrio and Como, where no contamination with GLY was detected, and Lecco, where GLY residues were detected only in one sample during the three years of the monitoring program, displayed lower values for the average use of herbicides, precisely 0.05 kg a.i./ha, 0.13 kg a.i./ha, and 0.01 kg a.i./ha, respectively. In the Varese province, no residues were detected, but the average herbicide use of 0.58 kg a.i./ha was estimated to be comparable to the values calculated for provinces with detected contaminations over the three years. The average use of herbicides in the remaining provinces within the Lombardy region ranged from 0.57 kg a.i./ha to 2.2 kg a.i./ha. Similarly, in the Emilia-Romagna region, the provinces with the lower average use were the ones where no contamination was detected over the three years; however, the values for average use calculated for the Forlì-Cesena province are comparable to the average herbicide use in provinces with different percentages of contamination (Table 2). The average use of herbicides in the provinces did not reflect the contamination rate. Multiple factors may be involved, not only the amount of the herbicides used but also, for instance, the frequency of use, the spatial distribution of treated crops, and the intensity of the agricultural activity developed in the considered territories.

### 3.3. Intensity of Agricultural Activity 

The intensity of agricultural activity may be representative of the pesticide use, as the percentage of the soil dedicated to the crops is potentially the subject of pesticide treatments and consequently may define the “density” of the pesticide use.

**Figure 1:**
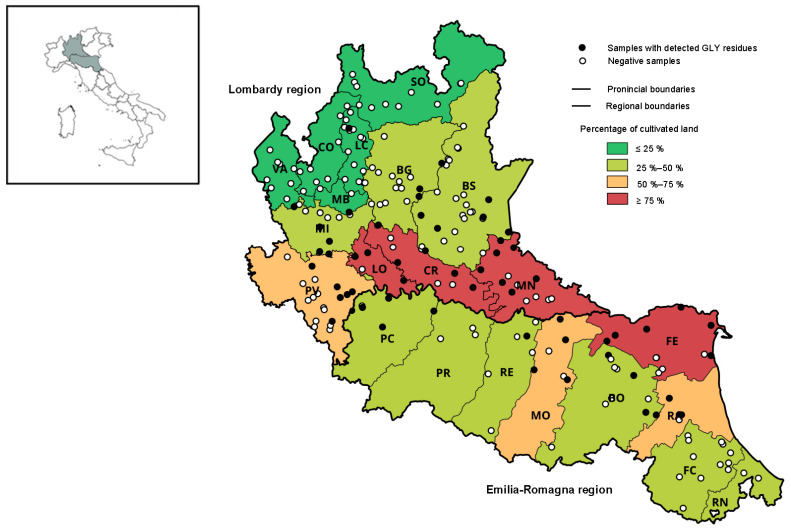
Graphical representation of the calculated land-use percentages dedicated to the agricultural practices in the provinces of the Lombardy and Emilia-Romagna regions and the location of the production sites of honey samples collected during the years 2020–2022. Black circles represent honey samples with detected GLY residues, and white circles represent samples with no detected GLY residues.

The considered Italian regions have slightly different percentages of overall cultivated areas, 45.1% for Lombardy and 52.7% for Emilia-Romagna. There are more-evident differences in the distribution of cultivated areas among provinces of each region. In nine provinces of Emilia-Romagna, the areas dedicated to cultivation range from around 38.2% and 80.2%, while in Lombardy, this distribution is wider, ranging from 7.5% to 90.8% (Figure 1), and different territorial arrangements may be observed. Zooming in on the agricultural arrangement of two regions, it can be noticed that in the Emilia-Romagna region, all provinces have more-uniform distributions of crop types, with 45% to 75% of cultivated areas dedicated to cereals, legumes, and forage crops. In Lombardy, the differentiation of crop types within the provinces is more pronounced. For instance, in the Sondrio province (mountain area), only 1.55% of agriculture area is dedicated to cereals, legumes, and forage crops, while Lodi, Milan, Pavia, Mantua, and Cremona reach above 80% of cultivated hectares dedicated to these types of crops.

**Figure 2:**
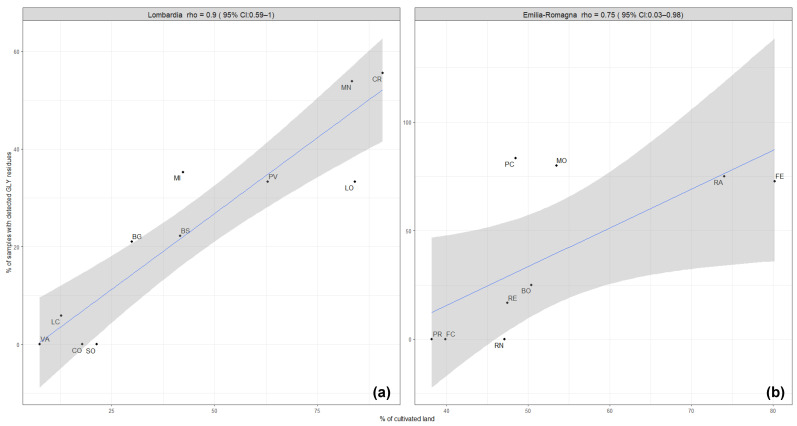
Correlation between the intensity of agricultural activity (% of cultivated land) and the occurrence of GLY residues in honey samples (% of samples with detected GLY residues) in the provinces of Lombardy (**a**) and Emilia-Romagna (**b**). The regression line is blue-colored, and the grey shadow represents the standard error.

In the Lombardy region, a correlation (rho coefficient = 0.9) was observed between the contamination of honey with GLY residues and the percentages of cultivated areas within the provinces (Figure 2a). In the provinces with less than 25% of hectares dedicated to cultivations (Varese, Sondrio, Como, and Lecco), no glyphosate residues (only one samples with detected GLY residues in Lecco) were detected during the monitoring period. These are provinces mainly located in the mountain area, where the agricultural sector is less developed. The Mantua and Cremona provinces are plain areas with highly developed agricultural activity, which displayed the highest contamination rate of honey samples with GLY residues in the monitoring program. In contrast, correlation was not observed in the Emilia-Romagna region (Figure 2b). This may be explained by the fact that the percentages of cultivated areas in the provinces of Emilia-Romagna are more uniform than in the Lombardy region, and the calculated contamination rates are based on insufficient amounts of monitoring data. 

**Figure 3:**
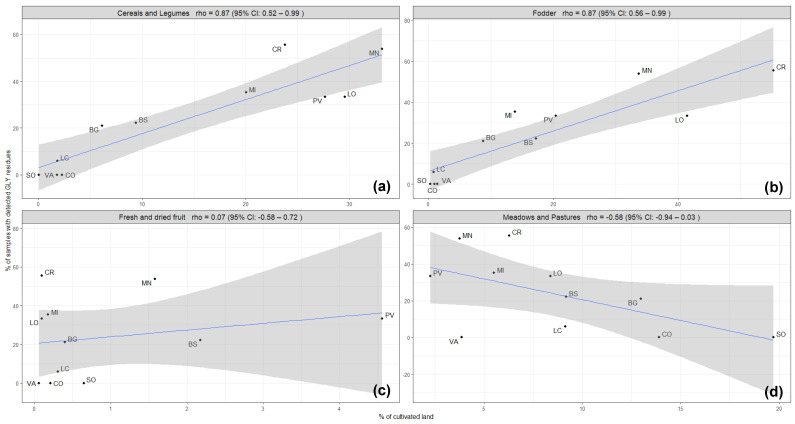
Occurrence of GLY residues in honey samples (% of samples with detected GLY residues) related to the intensity of different types of agricultural activities (% of cultivated land) developed in each province in the Lombardy region: (**a**) annual crops (cereals and legumes), (**b**) annual crops (fodder), (**c**) perennial crops (fresh and dried fruits), and (**d**) meadows and pastures. The blue line represents the regression line, and the grey shadow represents the standard error.

Therefore, further observations were made only for the Lombardy region, taking into consideration different types of crops cultivated in the provinces of the Lombardy region, particularly annual crops, such as cereals and legumes, fodder, perennial woody crops, and pastures and meadows. The correlation was conserved between the contamination rate of honey samples with GLY and the hectares dedicated to the annual crops, such as cereals and legumes (rho coefficient = 0.87) and fodder crops (rho coefficient = 0.87) (Figure 3), while no correlation was maintained with perennial crops (rho coefficient = 0.07) and pastures and meadows (rho coefficient = −0.58) (Figure 3). Annual crops are prevalent crop types cultivated in the Lombardy region, which may reflect the overall correlation. However, the frequency and the purposes of GLY use in annual crops are multiple compared to the perennial crops: in annual cropping systems, GLY is used for terminating cover crops before sowing, for weed control at pre-sowing, for pre-emergence or post-harvest stages, and for desiccation at pre-harvest stages, while in the woody perennial cropping systems, glyphosate use is usually limited to the control of weeds within the crop rows [12].

## 4. Discussion

The three-year monitoring results suggest that a low-level contamination of honey with glyphosate residues occurs in the two considered Italian regions. In Lombardy and Emilia-Romagna, GLY was detected in 23.8% (37/155) and 37.9% (25/66) of honey samples, respectively. EFSA reported lower percentages of quantifiable glyphosate residues in honey and apicultural products from European countries in 2018 (5.7%) and 2019 (6.8%) [36,37]. However, Bergero et al. tested honey from the Piedmont region in Italy for the presence of 67 pesticides, finding that the highest frequency was associated with GLY, resulting in 50% of samples with detectable residues of the herbicide [38]. 

Monitoring-data analysis based on the location of honey production sites in the provinces of the Lombardy region, where agricultural activities are developed at different levels, suggests that there may be a correlation between the honey contamination rate with glyphosate and the intensity of agricultural activity. Our results are preliminary and based on the monitoring data, although the contamination trends can be identified. Honey samples within the Lombardy region with more than 70% of the land dedicated to agricultural activity were steadily affected by glyphosate contamination through the monitoring years. In addition, in the Lombardy region, a widespread contamination of surface water bodies with glyphosate was reported, particularly in three areal clusters: the Monza–Brianza and North Milan province, Lodi and Pavia provinces, and Mantua province [39]. In this case, the authors suggest that the contamination was associated with both agricultural activity and urbanisation levels. Even though the reported water-body monitoring was performed in the 2008–2014 years, the same regional areas were the ones displaying the highest rate of honey samples with detected GLY residues in this study. A positive correlation between honey contamination with GLY and the proximity to the large-scale agriculture attributed through geospatial analysis was reported by Berg et al. [40]. In contrast, Medici et al. state that the configuration of the landscape surrounding the apiaries is not enough to explain the relationship with residues of GLY and its metabolite AMPA in honey [41]. 

Bees and hive products have a great potential to be evaluated as the sentinels of environmental pesticide pollution, and honey may represent a valuable matrix for tracing the hydrophilic-pesticide distribution in the environment and focus on the pathways involved in the contamination.

The trends observed in this work suggest a possible correlation of honey contamination with GLY and the extension of agricultural activity developed in the considered territories. However, the reported considerations are preliminary and based on a limited set of data, in which distribution through the monitoring years is not homogeneous due to the differences in the development of apicultural sectors within the provinces. More-specific and detailed studies are necessary to further understand at which extent the proximity to the cultivated areas, the agricultural practices, and the frequencies of the herbicide application may influence honey contamination with the herbicide.

## Figures and Tables

**Table 1:** Identification of samples with detected GLY residues (>LOQ) for each province in the Lombardy and Emilia-Romagna regions. The non-compliant concentrations exceeding the MRL of 0.05 mg kg^−1^ are in bold and marked with asterisks. The concentrations exceeding the MRL but identified as compliant due to the uncertainty calculation according to [28] are in bold.

	2020			2021			2022		
	Nr > LOQ	(mg kg^−1^)	Total	Nr > LOQ	(mg kg^−1^)	Total	Nr > LOQ	(mg kg^−1^)	Total
**Lombardy ^1^**									
BG	2	0.013	6	2	0.023	7	0		6
		**0.067**			**0.059**				
BS	1	0.018	11	3	0.016	8	2	0.041	8
					0.015			0.032	
					0.012				
CO	0		4	0		3	0		2
CR	1	0.04	3	3	0.25 *	4	1	0.018	2
					**0.072**				
					0.011				
LC	1	0.01	4	0		6	0		7
LO	0		2	1	**0.064**	2	1	0.024	2
MB	-	-	-	0		2	-	-	-
MI	2	0.017	5	2	0.023	5	2	0.011	5
		0.01			0.011			0.031	
MN	1	0.035	4	2	0.025	4	4	0.028	5
					0.035			0.013	
								0.032	
								0.049	
PV	4	**0.059**	6	0		6	2	0.011	6
		0.013						0.039	
		0.025							
		0.01							
SO	0		3	0		4	0		4
VA	0		1	0		4	0		4
**Total**	**12**		**49**	**13**		**55**	**12**		**50**
**Emilia-Romagna ^1^**									
BO	1	0.01	6	2	0.017	5	1	0.018	5
					0.024				
FC	0		3	0		4	0		5
FE	5	0.036	5	1	0.017	3	2	0.049	3
		0.011						0.013	
		0.31 *							
		0.01							
		0.024							
MO	2	0.012	2	1	0.02	1	1	0.024	2
		0.015							
PC	2	0.01	2	2	0.017	2	1	0.035	2
		0.03			0.017				
PR				0		2	0		1
RA	1	**0.062**	1	0		1	2	0.036	2
								0.022	
RE	1	0.032	3	0		2	0		1
RN				0		2	0		1
**Total**	**12**		**22**	**6**		**22**	**7**		**22**

^1^ Lombardy region: Bergamo (BG), Brescia (BS), Como (CO), Cremona (CR), Lecco (LC), Lodi (LO), Mantua (MN), Monza and Brianza (MB), Milan (MI), Pavia (PV), Sondrio (SO), and Varese (VA). Emilia-Romagna region: Bologna (BO), Ferrara (FE), Forlì-Cesena (FC), Modena (MO), Piacenza (PC), Parma (PR), Ravenna (RA), Reggio-Emilia (RE), and Rimini (RN).

**Table 2:** Contamination rates of honey samples with GLY residues and the average use of herbicides (kg a.i./hectare of cultivated land) in the provinces within the Lombardy and Emilia-Romagna regions. (n.d. = not detected).

Lombardy (Province)	% of Samples with Detected GLY Residues	kg a.i./Hectare of Cultivated Land	Emilia-Romagna (Province)	% of Samples with Detected GLY Residues	kg a.i./Hectare of Cultivated Land
BG	21.1	0.63	BO	25.0	0.71
BS	22.2	0.58	FC	n.d.	0.56
CO	n.d.	0.13	FE	72.7	1.90
CR	55.6	1.13	MO	80.0	1.33
LC	5.9	0.01	PC	83.3	1.23
LO	33.3	2.22	PR	n.d.	0.32
MI	40.0	0.73	RA	75.0	3.41
MN	53.9	0.57	RE	16.7	0.18
PV	33.3	1.72	RN	n.d.	0.07
SO	n.d.	0.05			
VA	n.d.	0.59			

## Data Availability

The data used to support the findings of this study can be made available by the corresponding author upon request.
